# Crystal structure and Hirshfeld surface analysis of an etoxazole metabolite designated R4

**DOI:** 10.1107/S2056989025008084

**Published:** 2025-09-19

**Authors:** Chaluvarangaiah Sowbhagya, Thaluru M. Mohan Kumar, Papegowda Bhavya, Hemmige S. Yathirajan, Sean Parkin

**Affiliations:** a Department of Physical Sciences, Amrita School of Engineering, Amrita Vishwa Vidyapeetham, Bengaluru-560 035, India; b Department of Applied Sciences, New Horizon College of Engineering, Bengaluru-560 103, India; c Department of Studies in Chemistry University of Mysore, Manasagangotri Mysuru-570 006 India; d Department of Chemistry University of Kentucky,Lexington KY 40506-0055 USA; Institute of Chemistry, Chinese Academy of Sciences

**Keywords:** etoxazole metabolite **R4**, insecticide, acaricide, Hirshfeld-surface analysis, crystal structure

## Abstract

A metabolite of the insecticide/acaricide etoxazole, designated **R4** {systematic name *N*-[1-(4-*tert*-butyl-2-eth­oxy­phen­yl)-2-hy­droxy­eth­yl]-2,6-di­fluoro­benz­amide, C_21_H_25_F_2_NO_3_}, is presented. The mol­ecular structure has a central *N*-(2-hy­droxy­ethyl)formamide group flanked by 4-*tert*-butyl-2-eth­oxy­phenyl and 2,6-di­fluoro­phenyl-substituted rings. The overall conformation is defined by its torsional degrees of freedom [N—C—C—C = 56.09 (18) and 99.41 (18)°], which place the 4-*tert*-butyl-2-eth­oxy­phenyl and 2,6-di­fluoro­phenyl rings at a dihedral angle of 70.66 (5)°. In the crystal, mol­ecules are linked by a strong O—H—O hydrogen bond into chains that extend parallel to the *a*-axis. There are also weaker C—H—F and π-stacking [centroid–centroid distance = 4.266 (2) Å] inter­actions. A Hirshfeld surface analysis reveals that the inter­molecular contacts are dominated by inter­actions involving hydrogen, the most abundant being H⋯H (54.1%), H⋯O/O⋯H (13.0%), H⋯F/F⋯H (12.8%), and H⋯C/C⋯H (12.8%).

## Abstract 2

The crystal structure and Hirshfeld surface analysis of *N*-[1-(4-*tert*-butyl-2-eth­oxy­phen­yl)-2-hy­droxy­eth­yl]-2,6-di­fluoro­benzamide, C_21_H_25_F_2_NO_3_, a metabolite of the insecticide/acaricide etoxazole designated **R4**, is presented.

## 1. Chemical context

The etoxazole metabolite designated **R4** [systematic name: *N*-[1-(4-*t*ert-butyl-2-eth­oxy­phen­yl)-2-hy­droxy­eth­yl]-2,6-di­fluoro­benzamide], C_21_H_25_F_2_NO_3_, which is found in metabolism studies of plants and rats, as well as in soil, is derived from etoxazole, an organofluorine chitin synthesis inhibitor. Etoxazole is a member of the oxazoline class of insecticides, having been developed as a new-generation insecticide and acaricide (Li *et al.*, 2014 ▸). It has been used globally since 1998 (Park *et al.*, 2020 ▸). A comprehensive review of the biological activity of oxazole derivatives was published by Kakkar & Narasimhan (2019 ▸), while Joshi *et al.* (2023 ▸) provided a detailed review of their chemistry. Recent research has also assessed the risks of oxidative stress and multiple toxicities induced by etoxazole (Macar *et al.*, 2022 ▸). The synthesis and activity of novel acaricidal/insecticidal 2,4-diphenyl-1,3-oxazolines were re­por­ted by Suzuki *et al.* (2002 ▸).
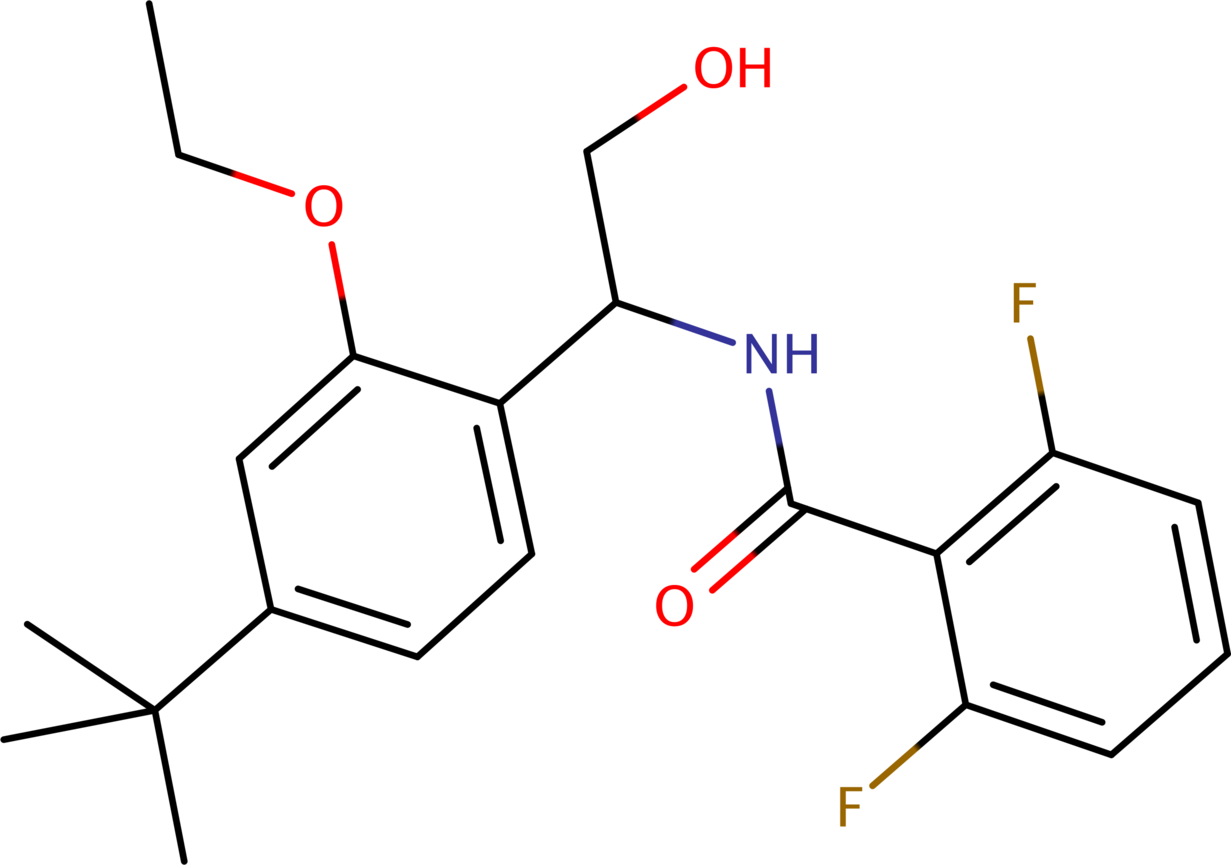


Several analogues of etoxazole and its precursors have been synthesized by Liu *et al.* (2013 ▸). We have recently reported the crystal structures of etoxazole (Sowbhagya *et al.*, 2025 ▸) and the etoxazole metabolite **R13** (Mohan Kumar *et al.*, 2024 ▸). In view of the agricultural importance of etoxazole and its metabolites, this paper reports the crystal structure and Hirshfeld-surface analysis of the etoxazole metabolite **R4**.

## 2. Structural commentary

**Figure 1:**
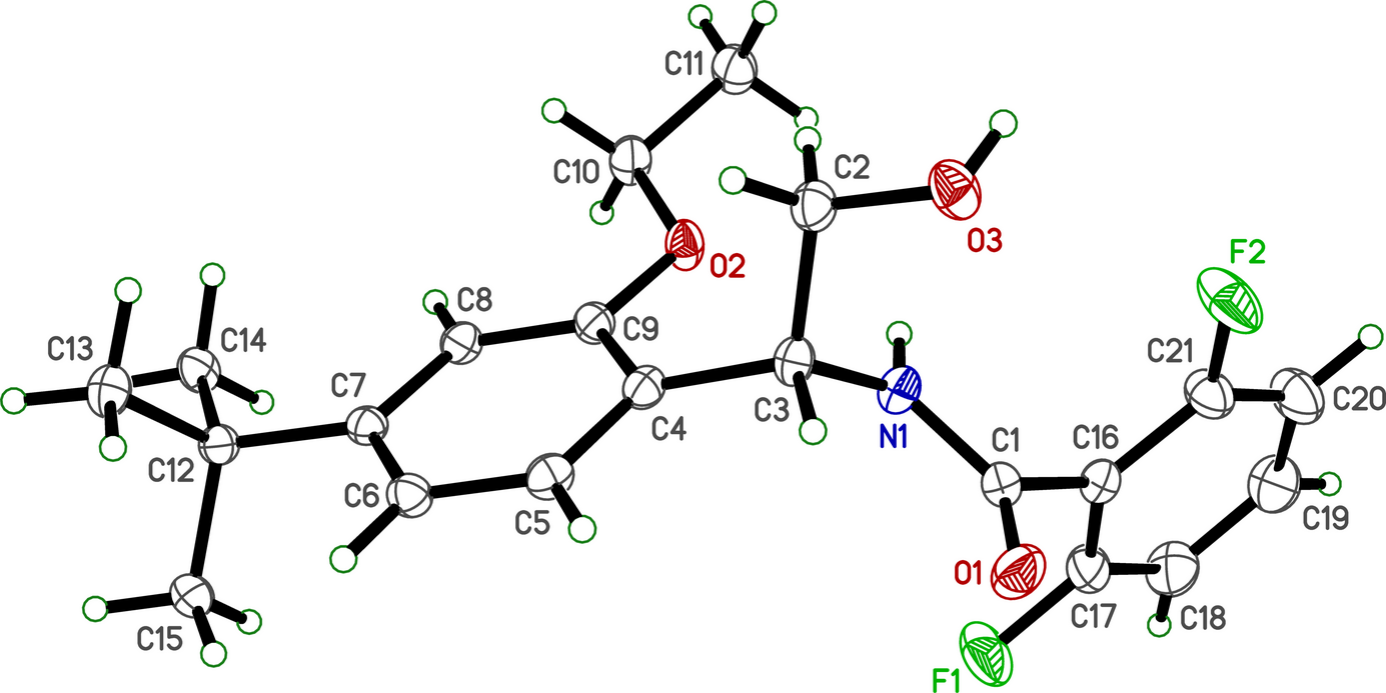
An ellipsoid plot (50% probability) of etoxazole metabolite **R4**. Hydrogen atoms are drawn as small circles of arbitrary radius.

The crystal structure of **R4** is monoclinic, having the symmetry of space group *Cc*, with a single mol­ecule in the asymmetric unit (*Z*′ = 1). The mol­ecular structure (Fig. 1 ▸) consists of a central (2-hy­droxy­eth­yl)formamide moiety flanked by 4-*tert*-butyl-2-eth­oxy­phenyl and 2,6-di­fluoro­phenyl substituted rings. Individual bond lengths and angles all fall within the normal ranges (Allen *et al.*, 1987 ▸).

The amide group (C1,O1,C16,N1,H1N,C3) is almost planar [r.m.s.d. = 0.0343 Å, largest = 0.049 (6) Å at N1]. The mol­ecular conformation is determined by torsion about the bonds connecting the rings to this central linker: N1—C3—C4—C9 [56.09 (18) Å] and N1—C1—C16—C17 [99.41 (18) Å]. These torsions lead to dihedral angles between the mean amide plane (excluding H1*N*) and the 4-*t*-butyl-2-eth­oxy­phenyl and 2,6-di­fluoro­phenyl rings of 49.84 (5)° and 82.74 (5)°, and a dihedral angle between the two benzene rings of 70.66 (5)°. Additional degrees of conformational flexibility serve to orient the eth­oxy group [C4—C9—O2—C10 = −164.82 (12)°], hy­droxy­ethyl [N1—C3—C2—O3 = −63.45 (15)°] and *tert*-butyl [C6—C7—C12—C13 = 60.97 (16)°] groups.

## 3. Supra­molecular features

**Figure 2:**
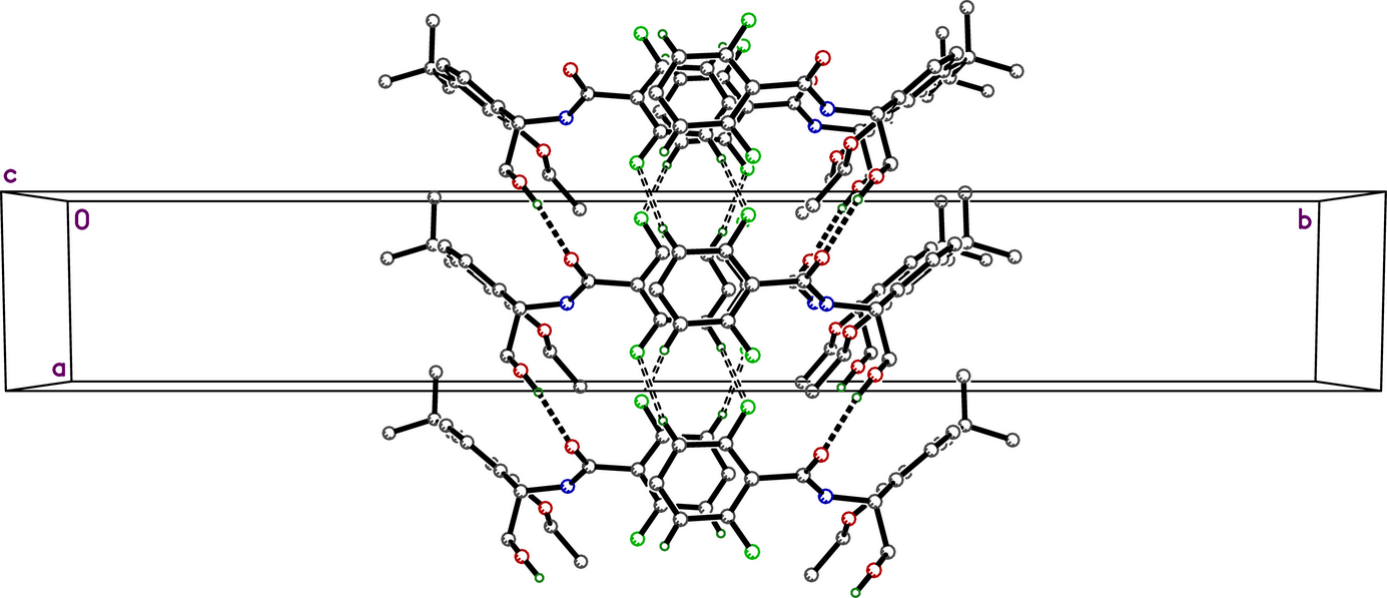
A partial packing plot of the **R4** crystal structure viewed down the *c*-axis. Conventional O—H⋯O hydrogen bonds are drawn as solid dashed lines, while weaker C—H⋯F contacts are drawn as open dashed lines.

**Figure 3:**
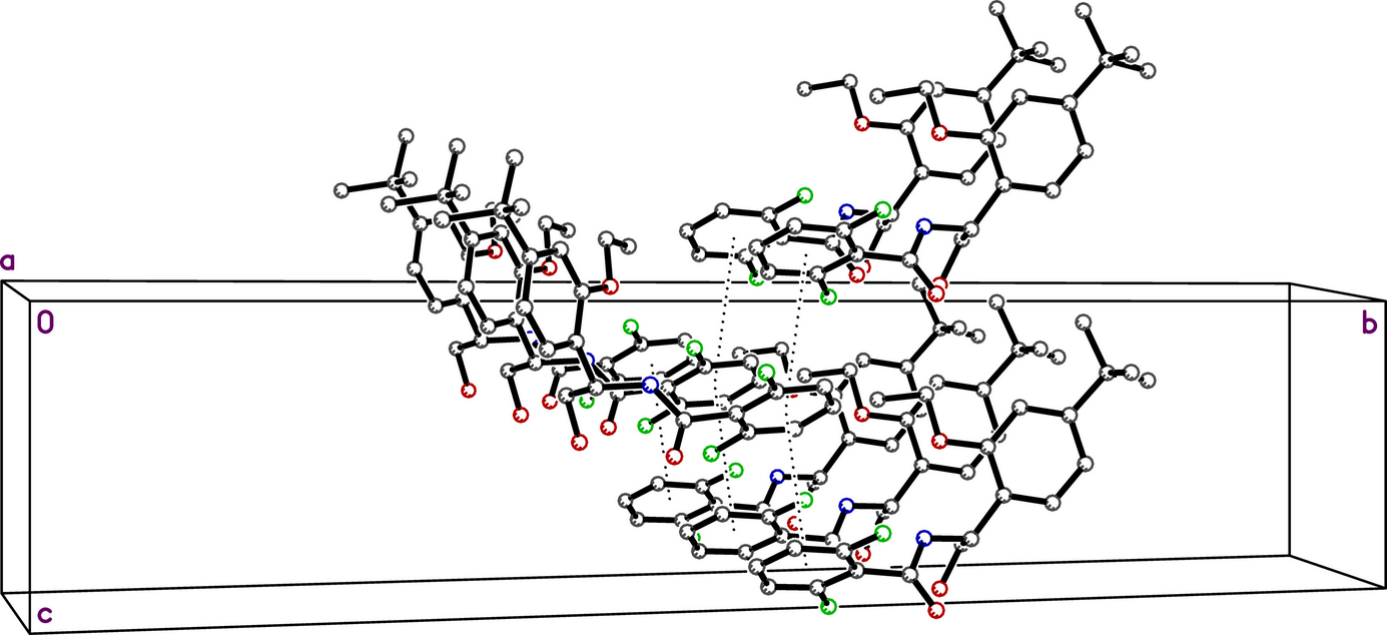
A partial packing plot of **R4** viewed approximately down the *a*-axis. Stacking of the di­fluoro­phenyl rings is highlighted by dotted lines joining adjacent ring centroids.

There is only one conventional hydrogen bond in the crystal structure of **R4**, *i.e*., O3—H3*A*⋯O1^i^ with *d_D⋯A_* = 2.8068 (17) Å, which links mol­ecules into chains that propagate parallel to the *a*-axis, as shown in Fig. 2 ▸. However, there are also weak hydrogen-bond like contacts between adjacent di­fluoro­phenyl rings: C18—H18⋯F2^ii^ [*d_D⋯A_* = 3.505 (2) Å], and C20—H20⋯F1^iii^ [*d_D⋯A_* = 3.473 (2) Å] (all symmetry codes are as per Table 1 ▸), forming ribbons roughly parallel to (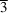,1,6) extending approximately along the [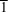,0,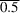], also shown in Fig. 2 ▸. These inter­actions are qu­anti­fied in Table 1 ▸. The di­fluoro­phenyl rings of glide-related (*x*, 1 − *y*, *z* + 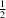) mol­ecules also stack into slightly zigzagged alternating columns parallel to the *c*-axis, as shown in Fig. 3 ▸, with centroid–centroid stacking distances of 4.266 (2) Å. The dihedral angles between adjacent rings in these stacks is 8.68 (8)°.

**Figure 4:**
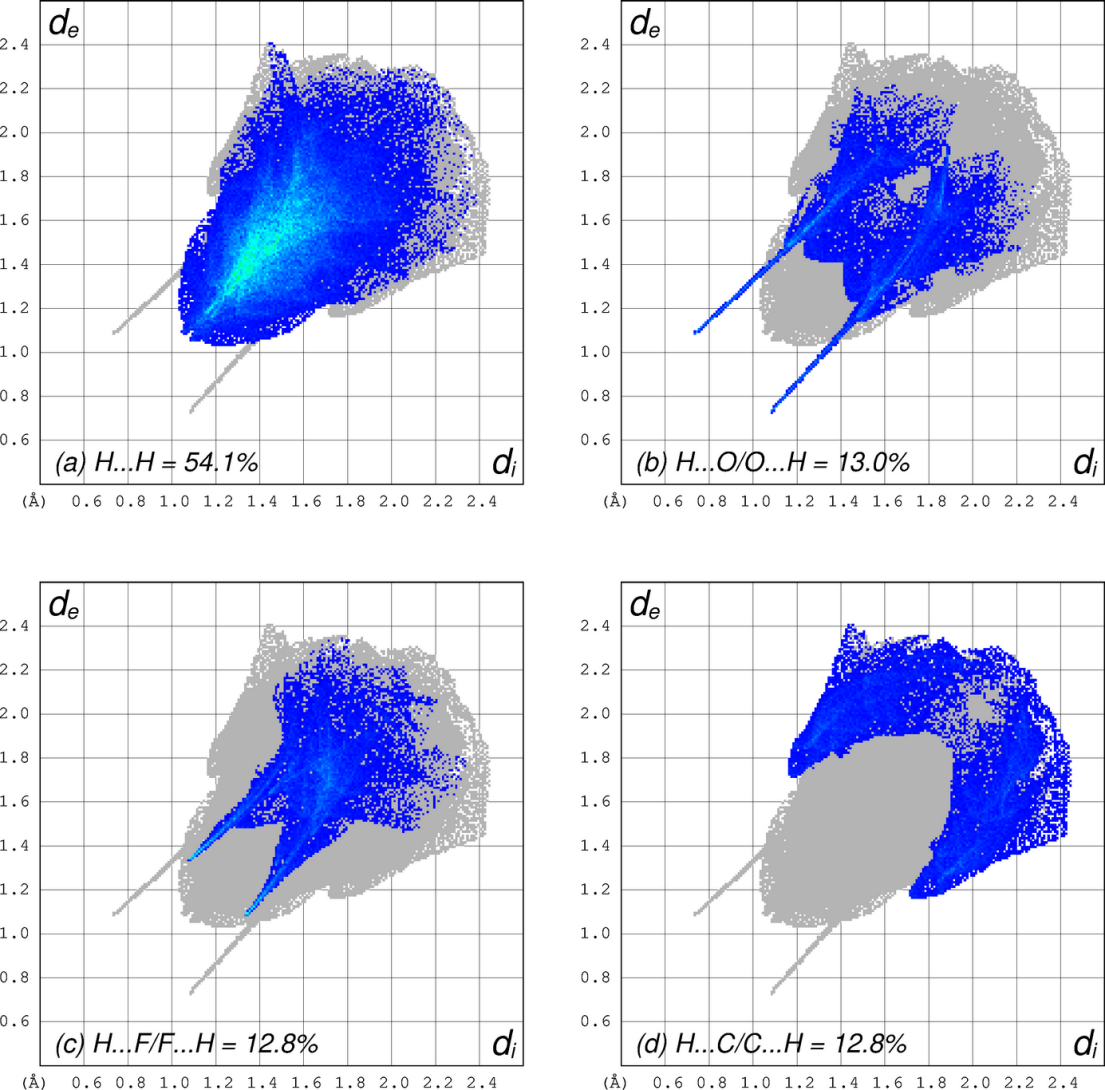
Two-dimensional fingerprint plots showing the most abundant types of atom–atom contacts present in the crystal packing: (*a*) H⋯H = 54.1%, (*b*) H⋯O/O⋯H = 13.0%, (*c*) H⋯F/F⋯H = 12.8%, and (*d*) C⋯H/H⋯C = 12.8%.

A Hirshfeld surface (HS) analysis performed using *CrystalExplorer* (Spackman *et al.*, 2021 ▸) indicates that the majority (92.7%) of inter­molecular contacts involve hydrogen. Two-dimensional fingerprint plots for H⋯H (54.1%), H⋯O/O⋯H (13.0%), H⋯F/F⋯H (12.8%), and H⋯C/C⋯H (12.8%) are shown in Fig. 4 ▸. All other types of inter­molecular contacts contribute 2.3% or less to the HS.

## 4. Database survey

A search of the Cambridge Structural Database [CSD, v6.00, April 2025; Groom *et al.*, 2016 ▸) on the core framework of **R4** but with the fluorine, hy­droxy­methyl, eth­oxy, and *tert*-butyl groups specified as ‘any’ returned 2593 hits. If the two fluorines were set to ‘any halogen’, then ten (nine unique) matches were found. A similar search fragment, but with the two fluorines in place gave six structures, five of which were unique. CSD entry CEDQIN (Valkonen *et al.*, 2010 ▸) is *N*-benzyl-2,3,4,5,6-penta­fluoro­benzamide. Structure NUCYOZ (Kaukorat *et al.*, 1996 ▸), or *N*-benzyl-*N*-(2,6-di­fluoro­benzo­yl)amino­methyl­dimethyl­phosphine oxide includes the di­fluoro­phenyl group of **R4**, but has benzyl and methyl-di­methyl­phosphine oxide attached at its N atom. Structure KOWTOJ (Puigcerver *et al.*, 2024 ▸) is a large urea-based rotaxane, while PUMHAH and PUMHEL (Arunachalam & Ghosh, 2009 ▸) are hexa­podal amides. These latter three structures have little in common with **R4**.

In addition to the above, crystal structures of etoxazole (DULGUQ: Sowbhagya *et al.*, 2025 ▸) and its metabolite **R13** (UGUQUM: Mohan Kumar *et al.*, 2024 ▸) were recently rep­orted. Lei *et al.* (2009 ▸) published the crystal structure of 2-(3-methyl-2-nitro­phen­yl)-4,5-di­hydro-1,3-oxazole (MOKMAB), an inter­mediate in the synthesis of anthranilamide insecticides. A crystal structure of ethyl 3-(4-chloro­phen­yl)-5-[(*E*)-2-(di­methyl­amino)­ethen­yl]-1,2-oxazole-4-carboxyl­ate (JADRIS), was described by Efimov *et al.* (2015 ▸), while the structure of 2,2-diphenyl-5-di­chloro­methyl­ene-3-oxazoline-4-ethyl­carb­oxyl­ate (KUXKIX), a diphenyl oxazoline compound, was reported by Puranik *et al.* (1992 ▸). Phenyl­pyrazole-based insecticide structures have been reported by Priyanka *et al.* (2022 ▸) and Vinaya *et al.*, (2023 ▸) (FERPOL and GIBJEK, respectively).

## 5. Synthesis and crystallization

The sample of **R4** was obtained as a gift from Honeychem Pharma Research Pvt. Ltd. It was purified by column chromatography and recrystallized from hexane by slow evaporation to yield colourless crystals.

## 6. Refinement

Crystal data, data collection and structure refinement details are summarized in Table 2 ▸. All hydrogen atoms were found in difference-Fourier maps. Those bonded to carbon were included in the refinement using riding models, with constrained distances set to 0.95 Å (C*sp*^2^—H), 0.98 Å (*R*CH_3_), 0.99 Å (*R*_2_CH_2_) and 1.00 Å (*R*_3_CH). The hydroxyl hydrogen was also treated as riding, but its bond distance was refined. The amide hydrogen atom coordinates were refined freely. *U*_iso_(H) parameters were set to values of either 1.2*U*_eq_ or 1.5*U*_eq_ (*R*CH_3_, OH, NH) of the parent atom.

## Supplementary Material

Crystal structure: contains datablock(s) I, global. DOI: 10.1107/S2056989025008084/nx2029sup1.cif

Structure factors: contains datablock(s) I. DOI: 10.1107/S2056989025008084/nx2029Isup2.hkl

Supporting information file. DOI: 10.1107/S2056989025008084/nx2029Isup3.cml

CCDC reference: 2487064

Additional supporting information:  crystallographic information; 3D view; checkCIF report

Additional supporting information:  crystallographic information; 3D view; checkCIF report

## Figures and Tables

**Table 1:** Hydrogen-bond geometry (Å, °)

*D*—H⋯*A*	*D*—H	H⋯*A*	*D*⋯*A*	*D*—H⋯*A*
O3—H3*A*⋯O1^i^	0.85	1.96	2.8068 (17)	173
C18—H18⋯F2^ii^	0.95	2.57	3.505 (2)	168
C20—H20⋯F1^iii^	0.95	2.56	3.473 (2)	162

Symmetry codes: (i) 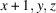; (ii) 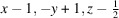; (iii) 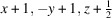.

**Table 2:** Experimental details

Crystal data	
Chemical formula	C_21_H_25_F_2_NO_3_
*M*_r_	377.42
Crystal system, space group	Monoclinic, *Cc*
Temperature (K)	100
*a*, *b*, *c* (Å)	5.7145 (1), 39.3575 (8), 8.4490 (2)
β (°)	95.500 (1)
*V* (Å^3^)	1891.50 (7)
*Z*	4
Radiation type	Mo *K*α
μ (mm^−1^)	0.10
Crystal size (mm)	0.16 × 0.08 × 0.04

Data collection	
Diffractometer	Bruker D8 Venture dual source
Absorption correction	Multi-scan (*SADABS*; Krause *et al.*, 2015 ▸)
*T*_min_, *T*_max_	0.916, 0.959
No. of measured, independent and observed [*I* > 2σ(*I*)] reflections	32266, 4721, 4583
*R*_int_	0.032
(sin θ/λ)_max_ (Å^−1^)	0.668

Refinement	
*R*[*F*^2^ > 2σ(*F*^2^)], *wR*(*F*^2^), *S*	0.025, 0.063, 1.05
No. of reflections	4721
No. of parameters	253
No. of restraints	2
H-atom treatment	H atoms treated by a mixture of independent and constrained refinement
Δρ_max_, Δρ_min_ (e Å^−3^)	0.18, −0.15
Absolute structure	Flack *x* determined using 2201 quotients [(*I*^+^)−(*I*^−^)]/[(*I*^+^)+(*I*^−^)] (Parsons *et al.*, 2013 ▸)
Absolute structure parameter	−0.04 (15)

Computer programs: *APEX5* (Bruker, 2023 ▸), *SHELXT* (Sheldrick, 2015*a* ▸), *SHELXL2019/3* (Sheldrick, 2015*b* ▸), *XP* in *SHELXTL* (Sheldrick, 2008 ▸), *SHELXTL* (Sheldrick, 2008 ▸) and *publCIF* (Westrip, 2010 ▸).

## supplementary crystallographic information

### N-[1-(4-tert-Butyl-2-ethoxyphenyl)-2-hydroxyethyl]-2,6-difluorobenzamide Crystal data

**Table:**

C_21_H_25_F_2_NO_3_	*F*(000) = 800
*M**_r_* = 377.42	*D*_x_ = 1.325 Mg m^−^^3^
Monoclinic, *C**c*	Mo *K*α radiation, λ = 0.71073 Å
*a* = 5.7145 (1) Å	Cell parameters from 9223 reflections
*b* = 39.3575 (8) Å	θ = 2.6–28.3°
*c* = 8.4490 (2) Å	µ = 0.10 mm^−^^1^
β = 95.500 (1)°	*T* = 100 K
*V* = 1891.50 (7) Å^3^	Cut block, colourless
*Z* = 4	0.16 × 0.08 × 0.04 mm

### N-[1-(4-tert-Butyl-2-ethoxyphenyl)-2-hydroxyethyl]-2,6-difluorobenzamide Data collection

**Table:**

Bruker D8 Venture dual source diffractometer	4721 independent reflections
Radiation source: microsource	4583 reflections with *I* > 2σ(*I*)
Detector resolution: 7.41 pixels mm^-1^	*R*_int_ = 0.032
φ and ω scans	θ_max_ = 28.4°, θ_min_ = 2.1°
Absorption correction: multi-scan (*SADABS*; Krause *et al*., 2015)	*h* = −7→7
*T*_min_ = 0.916, *T*_max_ = 0.959	*k* = −52→52
32266 measured reflections	*l* = −11→11

### N-[1-(4-tert-Butyl-2-ethoxyphenyl)-2-hydroxyethyl]-2,6-difluorobenzamide Refinement

**Table:**

Refinement on *F*^2^	Secondary atom site location: difference Fourier map
Least-squares matrix: full	Hydrogen site location: mixed
*R*[*F*^2^ > 2σ(*F*^2^)] = 0.025	H atoms treated by a mixture of independent and constrained refinement
*wR*(*F*^2^) = 0.063	*w* = 1/[σ^2^(*F*_o_^2^) + (0.0319*P*)^2^ + 0.425*P*] where *P* = (*F*_o_^2^ + 2*F*_c_^2^)/3
*S* = 1.05	(Δ/σ)_max_ = 0.001
4721 reflections	Δρ_max_ = 0.18 e Å^−^^3^
253 parameters	Δρ_min_ = −0.15 e Å^−^^3^
2 restraints	Absolute structure: Flack *x* determined using 2201 quotients [(*I*^+^)-(*I*^-^)]/[(*I*^+^)+(*I*^-^)] (Parsons *et al.*, 2013)
Primary atom site location: structure-invariant direct methods	Absolute structure parameter: −0.04 (15)

### N-[1-(4-tert-Butyl-2-ethoxyphenyl)-2-hydroxyethyl]-2,6-difluorobenzamide Special details

**Table:**

Experimental. The crystal was mounted using polyisobutene oil on the tip of a fine glass fibre, which was fastened in a copper mounting pin with electrical solder. It was placed directly into the cold gas stream of a liquid-nitrogen based cryostat (Hope, 1994; Parkin & Hope, 1998). Diffraction data were collected with the crystal at 100K.
Geometry. All esds (except the esd in the dihedral angle between two l.s. planes) are estimated using the full covariance matrix. The cell esds are taken into account individually in the estimation of esds in distances, angles and torsion angles; correlations between esds in cell parameters are only used when they are defined by crystal symmetry. An approximate (isotropic) treatment of cell esds is used for estimating esds involving l.s. planes.
Refinement. Refinement progress was checked using *Platon* (Spek, 2020) and by an *R*-tensor (Parkin, 2000). The final model was further checked with the IUCr utility *checkCIF*.

### N-[1-(4-tert-Butyl-2-ethoxyphenyl)-2-hydroxyethyl]-2,6-difluorobenzamide Fractional atomic coordinates and isotropic or equivalent isotropic displacement parameters (Å^2^)

**Table:**

	*x*	*y*	*z*	*U*_iso_*/*U*_eq_	
F1	0.10244 (19)	0.54111 (3)	0.65974 (15)	0.0408 (3)	
F2	0.82092 (19)	0.54312 (2)	0.97487 (15)	0.0397 (3)	
O1	0.3290 (2)	0.59371 (3)	0.93314 (15)	0.0302 (3)	
O2	0.71769 (19)	0.62031 (3)	0.43543 (12)	0.0212 (2)	
O3	0.9202 (2)	0.63295 (3)	0.94174 (13)	0.0250 (2)	
H3A	1.037 (4)	0.6195 (6)	0.9370 (4)	0.038*	
N1	0.5634 (2)	0.59924 (3)	0.73239 (15)	0.0194 (2)	
H1N	0.647 (4)	0.5885 (5)	0.667 (3)	0.029*	
C1	0.4443 (3)	0.58136 (4)	0.83255 (18)	0.0197 (3)	
C2	0.8474 (3)	0.64540 (4)	0.78773 (18)	0.0215 (3)	
H2A	0.947047	0.635476	0.709989	0.026*	
H2B	0.866100	0.670400	0.785862	0.026*	
C3	0.5898 (2)	0.63611 (3)	0.74138 (17)	0.0179 (3)	
H3	0.498334	0.644115	0.829372	0.021*	
C4	0.4911 (2)	0.65424 (3)	0.59172 (16)	0.0173 (3)	
C5	0.3322 (2)	0.68064 (4)	0.60233 (16)	0.0184 (3)	
H5	0.279190	0.685846	0.702732	0.022*	
C6	0.2485 (2)	0.69966 (4)	0.47053 (17)	0.0183 (3)	
H6	0.140387	0.717618	0.482167	0.022*	
C7	0.3215 (2)	0.69264 (4)	0.32149 (16)	0.0163 (3)	
C8	0.4776 (3)	0.66565 (3)	0.30836 (17)	0.0172 (2)	
H8	0.526510	0.660023	0.207261	0.021*	
C9	0.5625 (2)	0.64686 (3)	0.44178 (16)	0.0172 (3)	
C10	0.8387 (3)	0.61756 (4)	0.29405 (18)	0.0227 (3)	
H10A	0.901343	0.640002	0.266150	0.027*	
H10B	0.729145	0.609578	0.203843	0.027*	
C11	1.0372 (3)	0.59254 (4)	0.3269 (2)	0.0258 (3)	
H11A	1.120836	0.589981	0.231619	0.039*	
H11B	0.973612	0.570494	0.355537	0.039*	
H11C	1.146264	0.600885	0.414935	0.039*	
C12	0.2316 (2)	0.71494 (3)	0.17940 (16)	0.0165 (2)	
C13	0.3109 (3)	0.75181 (4)	0.21162 (17)	0.0214 (3)	
H13A	0.258401	0.765956	0.119536	0.032*	
H13B	0.482702	0.752639	0.230125	0.032*	
H13C	0.241988	0.760353	0.305750	0.032*	
C14	0.3229 (3)	0.70329 (4)	0.02407 (17)	0.0202 (3)	
H14A	0.259798	0.718142	−0.062863	0.030*	
H14B	0.272400	0.679858	0.001201	0.030*	
H14C	0.495007	0.704400	0.034257	0.030*	
C15	−0.0377 (2)	0.71376 (4)	0.15693 (18)	0.0216 (3)	
H15A	−0.095577	0.730599	0.077110	0.032*	
H15B	−0.100581	0.718903	0.258160	0.032*	
H15C	−0.089114	0.691042	0.121158	0.032*	
C16	0.4627 (3)	0.54335 (4)	0.81631 (19)	0.0204 (3)	
C17	0.2889 (3)	0.52419 (4)	0.7333 (2)	0.0267 (3)	
C18	0.2970 (3)	0.48934 (4)	0.7214 (2)	0.0327 (4)	
H18	0.174972	0.477132	0.661880	0.039*	
C19	0.4885 (3)	0.47258 (4)	0.7989 (2)	0.0330 (4)	
H19	0.498128	0.448527	0.792403	0.040*	
C20	0.6650 (3)	0.49042 (4)	0.8852 (2)	0.0330 (4)	
H20	0.794767	0.478893	0.939638	0.040*	
C21	0.6492 (3)	0.52529 (4)	0.8907 (2)	0.0261 (3)	

### N-[1-(4-tert-Butyl-2-ethoxyphenyl)-2-hydroxyethyl]-2,6-difluorobenzamide Atomic displacement parameters (Å^2^)

**Table:**

	*U*^11^	*U*^22^	*U*^33^	*U*^12^	*U*^13^	*U*^23^
F1	0.0317 (6)	0.0354 (6)	0.0509 (7)	−0.0035 (4)	−0.0197 (5)	0.0103 (5)
F2	0.0310 (5)	0.0247 (5)	0.0585 (7)	0.0000 (4)	−0.0204 (5)	−0.0013 (4)
O1	0.0325 (6)	0.0265 (5)	0.0342 (6)	0.0047 (5)	0.0164 (5)	0.0063 (5)
O2	0.0269 (5)	0.0203 (5)	0.0172 (5)	0.0079 (4)	0.0072 (4)	0.0038 (4)
O3	0.0270 (6)	0.0303 (6)	0.0169 (5)	0.0050 (5)	−0.0021 (4)	0.0001 (4)
N1	0.0233 (6)	0.0179 (6)	0.0173 (5)	−0.0010 (5)	0.0043 (5)	0.0011 (4)
C1	0.0181 (6)	0.0204 (6)	0.0202 (6)	0.0012 (5)	0.0002 (5)	0.0045 (6)
C2	0.0237 (7)	0.0217 (7)	0.0186 (6)	−0.0023 (6)	−0.0009 (5)	0.0025 (5)
C3	0.0225 (7)	0.0165 (6)	0.0147 (6)	−0.0002 (5)	0.0022 (5)	0.0006 (5)
C4	0.0182 (6)	0.0182 (6)	0.0155 (6)	−0.0023 (5)	0.0018 (5)	0.0010 (5)
C5	0.0200 (6)	0.0210 (6)	0.0146 (6)	−0.0013 (5)	0.0036 (5)	−0.0015 (5)
C6	0.0176 (6)	0.0188 (6)	0.0187 (6)	0.0018 (5)	0.0027 (5)	−0.0005 (5)
C7	0.0144 (6)	0.0176 (6)	0.0167 (6)	−0.0017 (5)	0.0005 (5)	0.0003 (5)
C8	0.0187 (6)	0.0198 (6)	0.0133 (6)	0.0003 (5)	0.0031 (5)	0.0000 (5)
C9	0.0175 (6)	0.0161 (6)	0.0182 (6)	0.0009 (5)	0.0028 (5)	0.0001 (5)
C10	0.0276 (8)	0.0221 (7)	0.0199 (7)	0.0068 (6)	0.0096 (6)	0.0025 (6)
C11	0.0274 (8)	0.0235 (7)	0.0275 (8)	0.0064 (6)	0.0076 (6)	0.0022 (6)
C12	0.0161 (6)	0.0183 (6)	0.0149 (6)	−0.0003 (5)	0.0011 (5)	0.0010 (5)
C13	0.0259 (7)	0.0194 (6)	0.0187 (7)	−0.0013 (5)	0.0002 (5)	0.0014 (5)
C14	0.0213 (7)	0.0246 (7)	0.0146 (6)	0.0028 (5)	0.0003 (5)	0.0013 (5)
C15	0.0176 (7)	0.0249 (7)	0.0220 (7)	0.0019 (5)	−0.0003 (5)	0.0042 (6)
C16	0.0208 (6)	0.0190 (6)	0.0215 (6)	−0.0014 (5)	0.0023 (5)	0.0043 (5)
C17	0.0231 (7)	0.0285 (7)	0.0274 (7)	−0.0034 (6)	−0.0036 (6)	0.0069 (6)
C18	0.0328 (9)	0.0282 (8)	0.0359 (9)	−0.0110 (7)	−0.0028 (7)	0.0015 (7)
C19	0.0378 (9)	0.0187 (7)	0.0426 (10)	−0.0031 (7)	0.0036 (7)	0.0015 (7)
C20	0.0294 (8)	0.0227 (7)	0.0456 (10)	0.0038 (6)	−0.0035 (7)	0.0036 (7)
C21	0.0220 (7)	0.0218 (7)	0.0333 (8)	−0.0016 (6)	−0.0042 (6)	0.0016 (6)

### N-[1-(4-tert-Butyl-2-ethoxyphenyl)-2-hydroxyethyl]-2,6-difluorobenzamide Geometric parameters (Å, º)

**Table:**

F1—C17	1.3557 (19)	C10—H10A	0.9900
F2—C21	1.3517 (18)	C10—H10B	0.9900
O1—C1	1.2251 (19)	C11—H11A	0.9800
O2—C9	1.3749 (17)	C11—H11B	0.9800
O2—C10	1.4405 (17)	C11—H11C	0.9800
O3—C2	1.4152 (18)	C12—C14	1.528 (2)
O3—H3A	0.85 (3)	C12—C15	1.5334 (19)
N1—C1	1.3356 (19)	C12—C13	1.5367 (19)
N1—C3	1.4601 (18)	C13—H13A	0.9800
N1—H1N	0.88 (2)	C13—H13B	0.9800
C1—C16	1.5068 (19)	C13—H13C	0.9800
C2—C3	1.531 (2)	C14—H14A	0.9800
C2—H2A	0.9900	C14—H14B	0.9800
C2—H2B	0.9900	C14—H14C	0.9800
C3—C4	1.5132 (19)	C15—H15A	0.9800
C3—H3	1.0000	C15—H15B	0.9800
C4—C5	1.389 (2)	C15—H15C	0.9800
C4—C9	1.3982 (18)	C16—C21	1.381 (2)
C5—C6	1.388 (2)	C16—C17	1.384 (2)
C5—H5	0.9500	C17—C18	1.376 (2)
C6—C7	1.3918 (19)	C18—C19	1.387 (3)
C6—H6	0.9500	C18—H18	0.9500
C7—C8	1.3984 (19)	C19—C20	1.379 (3)
C7—C12	1.5352 (18)	C19—H19	0.9500
C8—C9	1.3958 (19)	C20—C21	1.376 (2)
C8—H8	0.9500	C20—H20	0.9500
C10—C11	1.508 (2)		
			
C9—O2—C10	116.88 (11)	H11A—C11—H11B	109.5
C2—O3—H3A	109.5	C10—C11—H11C	109.5
C1—N1—C3	123.16 (13)	H11A—C11—H11C	109.5
C1—N1—H1N	119.4 (13)	H11B—C11—H11C	109.5
C3—N1—H1N	116.9 (14)	C14—C12—C15	107.80 (12)
O1—C1—N1	124.83 (13)	C14—C12—C7	112.58 (11)
O1—C1—C16	120.26 (13)	C15—C12—C7	109.65 (11)
N1—C1—C16	114.90 (13)	C14—C12—C13	108.45 (12)
O3—C2—C3	110.08 (12)	C15—C12—C13	109.00 (12)
O3—C2—H2A	109.6	C7—C12—C13	109.30 (11)
C3—C2—H2A	109.6	C12—C13—H13A	109.5
O3—C2—H2B	109.6	C12—C13—H13B	109.5
C3—C2—H2B	109.6	H13A—C13—H13B	109.5
H2A—C2—H2B	108.2	C12—C13—H13C	109.5
N1—C3—C4	113.38 (11)	H13A—C13—H13C	109.5
N1—C3—C2	110.07 (12)	H13B—C13—H13C	109.5
C4—C3—C2	111.93 (11)	C12—C14—H14A	109.5
N1—C3—H3	107.0	C12—C14—H14B	109.5
C4—C3—H3	107.0	H14A—C14—H14B	109.5
C2—C3—H3	107.0	C12—C14—H14C	109.5
C5—C4—C9	117.61 (12)	H14A—C14—H14C	109.5
C5—C4—C3	119.53 (12)	H14B—C14—H14C	109.5
C9—C4—C3	122.77 (12)	C12—C15—H15A	109.5
C6—C5—C4	121.93 (13)	C12—C15—H15B	109.5
C6—C5—H5	119.0	H15A—C15—H15B	109.5
C4—C5—H5	119.0	C12—C15—H15C	109.5
C5—C6—C7	120.58 (13)	H15A—C15—H15C	109.5
C5—C6—H6	119.7	H15B—C15—H15C	109.5
C7—C6—H6	119.7	C21—C16—C17	115.80 (13)
C6—C7—C8	118.13 (12)	C21—C16—C1	121.81 (14)
C6—C7—C12	119.16 (12)	C17—C16—C1	122.31 (14)
C8—C7—C12	122.71 (12)	F1—C17—C18	119.11 (15)
C9—C8—C7	120.90 (12)	F1—C17—C16	117.33 (14)
C9—C8—H8	119.6	C18—C17—C16	123.56 (15)
C7—C8—H8	119.6	C17—C18—C19	118.04 (16)
O2—C9—C8	123.15 (12)	C17—C18—H18	121.0
O2—C9—C4	116.02 (12)	C19—C18—H18	121.0
C8—C9—C4	120.83 (12)	C20—C19—C18	120.74 (15)
O2—C10—C11	107.99 (12)	C20—C19—H19	119.6
O2—C10—H10A	110.1	C18—C19—H19	119.6
C11—C10—H10A	110.1	C21—C20—C19	118.62 (16)
O2—C10—H10B	110.1	C21—C20—H20	120.7
C11—C10—H10B	110.1	C19—C20—H20	120.7
H10A—C10—H10B	108.4	F2—C21—C20	119.27 (14)
C10—C11—H11A	109.5	F2—C21—C16	117.50 (13)
C10—C11—H11B	109.5	C20—C21—C16	123.23 (15)
			
C3—N1—C1—O1	6.4 (2)	C9—O2—C10—C11	166.89 (13)
C3—N1—C1—C16	−172.76 (13)	C6—C7—C12—C14	−178.45 (12)
C1—N1—C3—C4	−120.42 (15)	C8—C7—C12—C14	2.52 (18)
C1—N1—C3—C2	113.35 (15)	C6—C7—C12—C15	−58.45 (16)
O3—C2—C3—N1	−63.45 (15)	C8—C7—C12—C15	122.51 (14)
O3—C2—C3—C4	169.50 (11)	C6—C7—C12—C13	60.97 (16)
N1—C3—C4—C5	127.51 (14)	C8—C7—C12—C13	−118.06 (14)
C2—C3—C4—C5	−107.25 (15)	O1—C1—C16—C21	−95.15 (19)
N1—C3—C4—C9	−56.09 (18)	N1—C1—C16—C21	84.0 (2)
C2—C3—C4—C9	69.15 (17)	O1—C1—C16—C17	81.4 (2)
C9—C4—C5—C6	−1.1 (2)	N1—C1—C16—C17	−99.41 (18)
C3—C4—C5—C6	175.52 (13)	C21—C16—C17—F1	179.55 (15)
C4—C5—C6—C7	0.3 (2)	C1—C16—C17—F1	2.8 (2)
C5—C6—C7—C8	1.1 (2)	C21—C16—C17—C18	−0.8 (3)
C5—C6—C7—C12	−178.00 (12)	C1—C16—C17—C18	−177.54 (16)
C6—C7—C8—C9	−1.7 (2)	F1—C17—C18—C19	−179.42 (16)
C12—C7—C8—C9	177.37 (13)	C16—C17—C18—C19	0.9 (3)
C10—O2—C9—C8	15.5 (2)	C17—C18—C19—C20	0.0 (3)
C10—O2—C9—C4	−164.82 (12)	C18—C19—C20—C21	−1.1 (3)
C7—C8—C9—O2	−179.42 (13)	C19—C20—C21—F2	179.99 (17)
C7—C8—C9—C4	0.9 (2)	C19—C20—C21—C16	1.2 (3)
C5—C4—C9—O2	−179.23 (13)	C17—C16—C21—F2	−179.10 (14)
C3—C4—C9—O2	4.31 (19)	C1—C16—C21—F2	−2.3 (2)
C5—C4—C9—C8	0.5 (2)	C17—C16—C21—C20	−0.3 (3)
C3—C4—C9—C8	−176.00 (13)	C1—C16—C21—C20	176.46 (16)

### N-[1-(4-tert-Butyl-2-ethoxyphenyl)-2-hydroxyethyl]-2,6-difluorobenzamide Hydrogen-bond geometry (Å, º)

**Table:**

*D*—H···*A*	*D*—H	H···*A*	*D*···*A*	*D*—H···*A*
O3—H3*A*···O1^i^	0.85	1.96	2.8068 (17)	173
C18—H18···F2^ii^	0.95	2.57	3.505 (2)	168
C20—H20···F1^iii^	0.95	2.56	3.473 (2)	162

Symmetry codes: (i) *x*+1, *y*, *z*; (ii) *x*−1, −*y*+1, *z*−1/2; (iii) *x*+1, −*y*+1, *z*+1/2.
